# The People with Arthritis Can Exercise (PACE) Program: A Qualitative Evaluation of Participant Satisfaction

**Published:** 2005-06-15

**Authors:** Leigh F Callahan, Britta Schoster, Andrea Meier, Thelma Mielenz, Lisa DiMartino

**Affiliations:** Thurston Arthritis Research Center; Thurston Arthritis Research Center, University of North Carolina at Chapel Hill, Chapel Hill, NC; School of Social Work, University of North Carolina at Chapel Hill, Chapel Hill, NC; Thurston Arthritis Research Center and Division of Physical Therapy, University of North Carolina at Chapel Hill, Chapel Hill, NC; Thurston Arthritis Research Center, University of North Carolina at Chapel Hill, Chapel Hill, NC. Ms Martino is now with the Durham Veterans Affairs Medical Center, Durham, NC

## Abstract

**Introduction:**

Developed by the Arthritis Foundation, People with Arthritis Can Exercise is a community-based exercise program for individuals with arthritis. This qualitative study was designed to assess participant satisfaction with the program and examine motivators and barriers to attending program classes.

**Methods:**

We conducted an 8-week randomized controlled trial of People with Arthritis Can Exercise among 347 participants residing in 18 urban and rural communities across North Carolina. Semistructured telephone interviews were conducted with 51 of the participants. Participants were asked about their overall satisfaction with the program. Motivating factors and barriers to attending the classes, including content, instructor, location, and schedule, were examined.

**Results:**

Of the 51 participants interviewed, 96% were female, with an average age in years of 67 (range, 32–90 years). Participants reported deriving considerable social support from exercising in a group with others who have arthritis. They identified two main factors that motivated them to continue participating in the exercise class: ability to work at their own pace during the class and confidence that they could do different kinds of exercise safely. Participants also reported that the instructor played a vital role in sustaining their motivation to exercise. Among the participants, noncompleters of the program reported arthritis-related illness or insufficient physical challenge as key barriers to class participation.

**Conclusion:**

This study suggests that a group exercise program for older adults with arthritis promotes a sense of social support and increases self-efficacy for exercise by allowing participants to work at their own pace.

## Introduction

Regular physical activity has emerged as an important component of a healthy lifestyle. People who exercise regularly live longer and are healthier than those who are sedentary (1-5). Arthritis often leads to decreased physical activity, which over time reduces joint mobility, strength, fitness, and exercise participation and increases the risk for development of coronary heart disease (6,7). In the past, people with arthritis were cautioned to rest and were discouraged from participating in exercise activities. However, this approach has changed over the last quarter of a century. Since 1975, study results have consistently indicated that moderate-intensity aerobic exercise is safe and physically and psychologically beneficial for people with arthritis (2,8-22). Although people with arthritis tend to be less fit than their peers without arthritis, studies have demonstrated that most people with arthritis can safely participate in appropriate conditioning exercise programs to improve cardiovascular fitness, muscle strength, psychosocial status, and functional status. According to *Physical Activity and Health: A Report of the Surgeon General* (23), regular moderate aerobic or resistance training exercise programs relieve symptoms and improve function in people with rheumatoid arthritis, osteoarthritis, or both.

One way of motivating people with arthritis to be more physically active is to encourage their participation in community-based group exercise classes (24). The Arthritis Foundation (AF) has developed two such programs, the AF Aquatic Program (AFAP) and the People with Arthritis Can Exercise (PACE) program (24). AFAP is a water-based program, and PACE is land based.

PACE was developed in 1987 and revised in 1999. PACE is targeted for adults who are not currently exercising regularly and allows for variation in course content and scheduling. The PACE program is offered at basic and advanced levels. At the basic level, class content consists of range-of-motion, gentle strengthening, balance, weight-bearing, breathing, and endurance exercises at a level appropriate for participants with functional limitations. All exercises can be performed in a standing or seated position to accommodate individuals with different limitations. In addition to exercises, instructors provide education in proper body mechanics, breathing and relaxation techniques, self-management behaviors, body awareness, and exercise principles. These components are included to decrease symptoms such as pain, fatigue, depression, and stress. Instructors are also encouraged to promote self-care and self-esteem using behavioral strategies such as verbal contracting, buddy systems, exercise diaries, and discussion of home exercise problems. For a more detailed description of the PACE program, see Boutaugh (24).

While PACE and AFAP have previously been evaluated in different settings and appear to be beneficial, they are not widely used; fewer than 1% of individuals with arthritis have enrolled in or taken these classes (24,25). Because of the documented efficacy of exercise for arthritis, clinical and public health practitioners are recommending participation in exercise or physical activity programs like PACE and AFAP. The low participation rates in these programs show that researchers and practitioners need to pay more attention to the potential barriers and motivators for people with arthritis to take part in group exercise activities.

We conducted an 8-week randomized controlled trial (RCT) of the basic-level PACE program among 347 participants residing in 18 urban and rural communities across North Carolina. The primary goal of the RCT was to assess the effect of PACE on key arthritis-related health outcomes; however, we also included a qualitative component in this multimethod study to 1) examine participant satisfaction with the program and 2) identify factors such as motivators and barriers that might need further exploration. Qualitative analysis allows for exploration of areas that cannot be addressed fully in quantitative studies. These findings may help guide the public health community in development, dissemination, and promotion of an appropriate and a suitable community-based group exercise program for older adults with arthritis.

## Methods

### Participants

In fall 2003, 347 individuals enrolled in an RCT of PACE in 18 urban and rural community sites across North Carolina. To be eligible, participants had to be 18 years or older, exercise fewer than three times per week, and have any type of self-reported arthritis or joint pain with moderate to severe limitation in joint motion, strength, or both. Individuals exercising 3 or more days per week for 20 minutes or more each day were excluded. Arthritis or joint pain and physical limitations were assessed during the enrollment process using the short-version Health Assessment Questionnaire Disability Index (HAQ-DI) (26). Participants were also asked to report on pain, fatigue, and stiffness using visual analog scales based on the Multidimensional HAQ scale (27).

The intervention group included 168 randomly assigned participants who received the basic-level PACE class in the fall of 2003. Control subjects (n = 155) received a delayed treatment, participating in PACE classes after the initial 8-week intervention was completed. Twenty-four participants were not randomized because of transportation and other personal reasons. The class met two times a week for 8 weeks. For the purpose of the qualitative evaluation, participants in the intervention arm were classified into two groups based on class attendance: completers and noncompleters. Completers were participants who attended 75% or more of all classes, and noncompleters were participants who attended fewer than 75% of all classes.

A purposive sample comprising two completers and one noncompleter randomly chosen from each of the 18 PACE sites was selected for telephone interviews, with a sampling goal of 54 interviews (36 completers and 18 noncompleters). Fifty-one participants were actually interviewed (36 completers and 15 noncompleters). Completers were oversampled to obtain more information from participants who had the most exposure to the course. Table 1 details the health status and demographic characteristics of these interviewed participants.

### Interviews

All interviews were conducted by one of the researchers (LD) between October 2003 and February 2004 within a month after the participant completed the 8-week PACE exercise class. Participants were first called at home during the weekday, and if they could not be reached, they were contacted in the evening.

A semistructured interview guide was developed to elicit participant views on the factors that motivated them to attend PACE exercise classes and the barriers that prevented them from attending classes, including course content, the PACE instructor, and the class location and schedule. (See the Appendix for sample interview questions.) The order of the interview questions varied slightly depending on how the conversation developed during each interview; participants were only asked questions about the topics that they had not covered in their responses to earlier questions during the course of the interview. Interviews conducted with the noncompleters tended to be shorter than those conducted with the completers because the noncompleters had participated in significantly fewer classes and so could not comment as extensively. As certain themes arose, the researcher probed to explore these themes further and recorded notes of her impressions after each interview.

Interviews lasted an average of 17 minutes (range, 4–38 minutes). Digital audio files of the interviews were saved under the participant identification number to ensure confidentiality. The University of North Carolina School of Medicine Institutional Review Board approved all methods.

### Theoretical perspective

In this study, the information-motivation-behavioral skills (IMB) model was used to provide the conceptual framework for analyzing the factors that lead to exercise behavior. The model's major components were exercise information, exercise motivation, and exercise behavioral skills. The IMB model was originally developed in 1992 to predict HIV-preventive behavior; however, its concepts can be broadly applied to predict positive health behavior change in a range of contexts, such as exercise behavior (28).

### Data analysis

Two of the researchers (LD and BS) conducted the analyses, beginning with a verbatim transcription of the interviews. Once all data were transcribed, a random sample of 10 (20%) of the total transcripts was reviewed for accuracy and completeness by a member of the research team who had not been involved in the data collection process.

The data were then analyzed using NUD*IST (N6) (QSR International, Melbourne, Australia), a software program for qualitative data analysis. The initial categories were developed deductively based on the broad topics of each interview question, which measured the IMB model concepts. Subcategories were added to each of these initial base categories to further organize participant responses. After the initial round of coding was completed, the researchers reread the transcripts and condensed the list of deductive codes, retaining only those that occurred most frequently across all interviews.

The method of *constant comparison* was used to develop higher level themes (29). Transcripts were reread, and a series of inductive codes was created based on emerging themes. When new themes arose, all researchers were consulted to ensure consistent coding of the transcripts. Each time a new theme emerged, all transcripts were reread and recoded according to the new understandings. Key phrases used by the participants in the interviews were retained to name some of the inductive codes. The codes were eventually reduced and refined into key themes informed by the concepts from the IMB model (Table 2).

Baseline demographic and health status characteristics of the 51 qualitative participants were examined. Two-sample *t* tests for continuous variables and the Fisher exact test for categorical variables were used to assess differences between the completers and noncompleters.

## Results

Of the 51 participants interviewed, 96% were female, with an average age of 67 years (range, 32–90 years for the completers and 34–77 years for the noncompleters). Sixty-four percent of completers and 67% of noncompleters resided in urban areas. Completers attended an average of 75% of classes, whereas noncompleters attended an average of 13% of classes. Table 1 shows additional demographic and health status characteristics of the participants. Noncompleters reported arthritis-related illness or insufficient physical challenge as key barriers to participating in PACE. Completers reported missing class because of personal or family illness. One noncompleter was unable to attend because of lack of transportation, and many completers missed one to two classes because of scheduling conflicts. Both groups found the social support they received from the instructor and from the other class members to be a major motivational factor to participation in PACE. In addition, being able to work at their own pace during the class and feeling confident that they could do different exercise activities safely also played an important role in sustaining their motivation to exercise.

The components of the IMB model were used to organize the results of the thematic analyses. The IMB components adapted for this study were exercise information, exercise motivation, exercise behavioral skills, moderating factors (or barriers), and exercise behavior. The quotations presented in the text that follows were extracted from the interview transcripts to illustrate each of these themes. The term *participants* encompasses both completers and noncompleters.

### Themes related to exercise information

**Keep moving.** Participants described the overall information they received at the PACE exercise class as helpful and felt certain that even if it was not helpful to them, that it was helpful to other class members. Participants predominately talked about the importance of keeping their bodies in motion throughout the day. They perceived this as an effective strategy for staying active and preventing or reducing arthritis pain and stiffness. Many participants spoke of the informational brochures about arthritis and physical activity that they used to guide their exercise at home. They also used information on breathing and relaxation exercises to manage pain and stress:

Move. Just simply move. Do not overexert [or] hurt yourself, but absolutely do not be a "dottle-twee" and just don't move. And that means everything from when I sit and watch TV and do my finger exercises.

**Practical information.** Both completers and noncompleters regarded learning to move safely as an important topic. Participants' examples of safe movement included learning how to get up from a fall and transferring from one position to another. Many participants reported that these skills helped to quell their uncertainties about their arthritis and take control of their health by preparing for the future. Some participants spoke of wanting to maintain their independence as long as possible, so staying active and learning to move safely was essential. Learning such skills can enhance participants' confidence that they will be able to cope with and adjust to their arthritis. In turn, this positive attitude may also increase their self-esteem and sense of efficacy about being able to do what they want to do independently:

. . . If old people sit down, they'll get to a point where they can't get up. So you know I just want to keep movin' and doin' somethin' so I can continue to take care of myself. 'Cause I do live alone and I try to do what I can . . . so I don't have to call the children in to do it for me if I can help it.

### Themes related to exercise motivation

**Class social support*.*** A major motivator for both completers and noncompleters participating in the PACE class was social support received from other class participants and instructors. The classes provided supportive environments for the participants. Many participants mentioned that simply knowing that each week the group was expecting them to show up for class, as well as having a structured time set aside for exercise, was significant motivation to attend class:

Yeah, I'm much more likely to exercise if I've got motivation like that. "Okay, the rest of the crowd's comin', I better go, too." And that way you get to visit with everybody, too.

The group structure motivated participants not only to attend class but also to challenge themselves to move their bodies in ways they may not have if they had been exercising on their own:

Yeah that was challenging. That was interesting and challenging just to see what you can do, you know. It shows you that you can really do things I think was the best part of the class. You'd move things you thought you couldn't move before because everyone else was doing it with you [laughs].

Participants also valued being able to exercise in a group with others who lived with arthritis. Many commented about this aspect of the class, seeing it as an opportunity to interact and empathize with other people who could truly relate to them. It was also a time for sharing practical information, such as recommendations for a rheumatologist or arthritis-appropriate devices to use in the kitchen:

And I also enjoyed saying, "Dang man, this hurts today."  And they say, "Yeah, it does."  You know, just to have somebody else be in your shoes.

**Instructor support*.*** The instructors' personality characteristics were important factors in participants' perceived sense of support. Above and beyond all other topics, completers and noncompleters talked about how much they liked their instructors. When describing their instructors' personalities, they used words such as "nice," "patient," "friendly," and "polite." Participants' high regard for their instructors increased their desire to attend the class and helped them feel safe engaging in the recommended class exercises:

She is a very pleasin', talkin' person to you. When she [the instructor] exercise, she put a little somethin' in it. . . . She has a kind voice, and she makes a good instructor I think [laughs].

Participants frequently mentioned empathy as an important characteristic of a supportive instructor. In this context, empathy connotes the ability of the instructor to truly understand what it feels like to live with arthritis:

Well, I liked the fact that she herself had arthritis. It's not like getting someone who's never experienced any pain with arthritis telling you, "You can do this." I mean, she definitely said that if you feel pain, you can stop. And I thought that was very good.

Participants also frequently described instructor behaviors that they found supportive. These included paying personal attention to class members, skillfully demonstrating class exercises, looking up answers to participants' questions outside of class, and competently understanding and suggesting appropriate exercises for arthritis. Nearly all participants, both completers and noncompleters, talked about how the instructors paid personal attention to the class members, learning their names and calling them at home to check on them if they missed a class.

Participants appreciated being able to trust that their instructors would know what exercises were safe for them to perform. They liked being able to ask the instructors for modification suggestions when an exercise proved too difficult or uncomfortable. If a participant found that a particular exercise was too difficult or too painful to perform, the instructor would suggest modifications, such as assuming a sitting rather than a standing position:

She noticed each person, and she could tell what each person was goin' through in the body as they exercised. She'd call you out by name, and she says, "Looks like you're havin' pains. If you are, slow it down!" She was good.

Participants also appreciated when the instructors demonstrated the exercises and performed them along with the class:

And they did the exercises right along with us, and they showed us what we needed to do with our bodies. It was very . . . we could just mirror what they were doing, and it made it so much easier.

Completers and noncompleters generally reported only positive things about their instructors. The few negative comments included a participant feeling that she knew more about arthritis than her instructor and that her instructor did not do a good job of pacing the class. Another participant did not like that her instructor consulted the PACE book of exercises while performing the exercises with the group, perceiving the instructor as ill-prepared to teach the class. However, another participant in the same class liked this teaching method, reporting that it made him feel like they "were all learning to exercise together." Lastly, one participant complained that she felt the class was more about socializing than exercising and suggested that the instructor stick to a tighter exercise schedule. These comments were made equally by completers and noncompleters.

**I could do it.** Knowing that they could work at their own pace and modify the activities as needed, coupled with the strong sense of trust they had in their instructors, participants felt comfortable engaging in the exercises and trying things they had believed they could not do, both at home and in class. This increased their sense of self-efficacy, allowing participants with diverse functional capacities to participate in the class, tailor the level of difficulty according to their individual needs, and incorporate these exercise skills into their daily lives:

. . . It challenged me to try to get past the stiffness and pain . . . to just start loosening up. And I saw a lot of benefits from doing that. So that's what I'm trying to do now.

### Themes related to exercise behavioral skills

**At your own pace.** Instructors encouraged participants to work at their own pace, reminding them that exercise does not have to be aerobic and fast. The ability to exercise at one's own pace was one of the most frequently reported themes and was referred to by both the completers and the noncompleters:

I learned a lot taking it because that one little word, "at your own pace," it kinda clicked in my mind, you know, and just hearin' her say, "you can do it, at your own pace." You know, so she's sayin' you don't have to be rushin'. If you can't do it, don't do it, you know. That little word, "own pace."

### Themes related to moderating factors (barriers)

Completers and noncompleters differed in reporting barriers to participating in PACE.

**Personal illness.** A major factor affecting participants' motivation to participate in PACE was personal illness. Noncompleters often reported missing class because of arthritis-pain–related illness, whereas completers missed because of illness in general, such as being sick with the flu. Noncompleters explained that they could not predict how they would be feeling the day of the class; if their arthritis were to "flare up," it would make it nearly impossible for them to drive to class, and it would be uncomfortable to exercise. Only one noncompleter reported that exercising actually made his bodily pain worse.

**Class complaints.** Some noncompleters complained that their classes were not challenging enough. A few saw themselves as either significantly younger, more fit, or both, making it hard to relate to the other class participants:

I think I was in the wrong age bracket. There were very, very elderly people around me that couldn't even lift their arms, and I felt I was in the wrong place.

A few completers also commented on lack of class challenge and therefore should have been advised to enroll in the advanced-level PACE class.

### Themes related to exercise behavior

**Practice at home.** Participant reports support the IMB model predictions that the availability of exercise information and increased motivation to exercise affect exercise behavior either directly or indirectly through the acquisition of exercise skills. Both completers and noncompleters reported practicing the PACE exercises at home. One participant could not attend the class because she had to leave to care for an ill family member. Although she did not attend class, she used the pamphlets given to her by her instructor to guide her exercises. In this case, appropriate exercise information led directly to engagement in exercise behavior.

**Continue to exercise over time.** Completers and noncompleters who reported practicing the PACE exercises at home also reported that they continued to exercise after the PACE class had ended. In the class, they had developed the skills and confidence they needed to safely engage in an exercise routine at home. Noticing an improvement in level of pain and stiffness as a result of the exercise class encouraged participants to continue to exercise.

When you can do anything it makes you feel better. 'Cause I was doin' it everyday here at home, and I'm still doin' it. And I'm goin', "Hey, you know, this is great."

## Discussion

This qualitative evaluation contributes to our understanding of the suitability of PACE by allowing us to hear directly from the PACE participants and gain insight into the kinds of experiences PACE classes provided for those who enrolled in the program. These findings may serve as a model for future development of the PACE program in communities.

Results of this study did not reveal notable differences in the factors that motivated completers and noncompleters to participate in PACE classes. Interestingly, the differences between the groups were in the context of barriers; noncompleters generally missed class because of arthritis-related illness or insufficient physical challenge, and completers missed class because of personal or family illness. Participants emphasized the important roles of social support and self-efficacy in maintaining physical activity. PACE may enhance participants' beliefs in their ability to exercise by providing a supportive environment that allows them to modify the exercises as needed and to work at their own pace.

These findings were based on a group of mostly older adults with arthritis who reside in urban and rural areas across North Carolina. Participant responses, therefore, are specific to the experiences of older adults with arthritis, who may have different expectations for and perceptions of physical activity than younger people with arthritis. Although telephone interviews may have limited the depth of responses to the interview questions, they had the advantage (compared with in-person interviews) of enabling the researchers to contact a large number of participants in a relatively short period. To increase trustworthiness and internal validity, two researchers were involved in the initial data analysis. Both completers and noncompleters from each of the PACE sites were interviewed, thereby reducing potential selection bias. The large sample size of completers (two per site) helped to yield thematic saturation, thereby increasing the likelihood that the findings represented a comprehensive description of the experiences of these individuals. However, only one noncompleter was sampled at each site, resulting in an insufficient number of interviews to fully examine variations in their attitudes. Because of time and resource constraints, only the intervention, not the control group, was interviewed, which may have limited the depth of the findings.

This study is descriptive and intends to lay the groundwork for a future, more in-depth examination of the myriad factors that may affect a person's satisfaction with the PACE program. Because both completers and noncompleters most often remarked upon the importance of support derived from their instructors and class members when talking about their experiences in PACE, the role of social support as a motivating factor to engage in PACE merits further examination. Special attention should be placed on methods of recruiting and training the instructors, as the instructor role was key in sustaining the participants' motivation to exercise. Additionally, a more comprehensive understanding of the participant experiences in PACE could be achieved by analyzing participant demographic characteristics in relation to their qualitative responses. It is likely that factors such as urban or rural residence, functional status, age, and level of education affect a person's expectations and motivation to participate in an arthritis exercise program.

Promoting physical activity is a key public health strategy for addressing arthritis self-management. The results of this evaluation support the promotion of PACE as an appropriate and a desirable program for older adults with arthritis. The public health community can use this information to raise awareness of PACE and to encourage clinicians to recommend the program to their patients.

## Figures and Tables

**Table 1:** Demographic and Health Status Characteristics of Interviewed Participants (N = 51) Enrolled in the People with Arthritis Can Exercise (PACE) Study, North Carolina, 2004

**Characteristic**	**Completers (n=36)** **Mean (SD)**	**Noncompleters (n=15)** **Mean (SD)**	***P***
Age, years	70 (13)	60 (12)	.02
HAQ-DI^a^	1.0 (0.6)	1.2 (0.6)	.30
VAS pain scale^b^	47 (25)	52 (25)	.40
VAS fatigue scale^b^	49 (31)	58 (37)	.40
VAS stiffness scale^b^	44 (26)	61 (31)	.05

	**No. (%)**	**No. (%)**	

Female	33 (94)	15 (100)	.99
Nonwhite	8 (23)	3 (21)	.99
≤ High school	16 (46)	4 (27)	.30
Fair or poor general health	13 (37)	5 (33)	.99

a aHAQ-DI indicates the Health Assessment Questionnaire Disability Index. The score ranges from 0–3, with 0 meaning not disabled and 3 meaning completely disabled.

b VAS indicates visual analog scale. The VAS pain scale ranges from 0–100, with 0 representing no pain and 100 representing severe pain. The VAS fatigue scale and VAS stiffness scale also range from 0–100 with similar verbal anchors.

**Table 2:** Key Themes and Definitions Used in the People with Arthritis Can Exercise (PACE) Qualitative Analysis, Information-Motivation-Behavioral Skills Model, North Carolina, 2004

**Theme**	**Definition**

**Exercise information**	

Keep moving	Importance of continually moving your body
Practical information	Skills for safe movement, information about rheumatologists and arthritis aids and devices

**Exercise motivation**	

Class social support	Support from class members
Instructor support	Support from instructor
I could do it	Ability to do a particular exercise or feeling confident in the class in general

**Exercise behavioral skills**	

At your own pace	Ability to work at own pace when exercising

**Moderating factors (barriers)**	

Personal illness	Class missed because of personal illness
Class complaints	Class missed because of insufficient physical challenge

**Exercise behavior**	

Practice at home	PACE exercises practiced at home during course
Continue to exercise over time	PACE exercises continued after class ended

## Appendices

### Appendix: Sample Interview Guide Questions for Participants in People with Arthritis Can Exercise (PACE), North Carolina, 2004

**When you missed a PACE class, what was your reason for missing?**

Probe reasons:

- Transportation
- Illness/injury/pain/stiffness
- Didn’t feel like going
- Other commitments (e.g., doctor appointment)
- Forgot

**What was the main factor that encouraged you to attend the PACE exercise classes?**

Probe motivations:

- See friends at the class
- Exercise makes me feel good
- An opportunity to meet new people
- Learn more about my arthritis or joint pain

**How challenging were the PACE exercises for you?**

Follow-up:

- What parts of the class made them most challenging?
- What could have been done to make the exercise classes more challenging than they were?

**Tell me about some of the strengths and weaknesses of your PACE instructor.**

Probe:

- Can you remember any specific things that the instructor did or said during the PACE classes, or even between the PACE classes, that you particularly liked or disliked?

**Overall, how satisfied do you feel with the things you learned about living with your arthritis?**
